# Neural Signatures of Flattened Emotional Experience in Patients With Early Multiple Sclerosis: A Bayesian Approach

**DOI:** 10.1002/brb3.70987

**Published:** 2025-10-29

**Authors:** Torsten Wüstenberg, René Gieß, Judith Bellmann‐Strobl, Hagen Kunte, Friedemann Paul, Thomas D. Hälbig

**Affiliations:** ^1^ Core Facility for Neuroscience of Self‐Regulation (CNSR), Field of Focus 4 (FoF4) Heidelberg University Heidelberg Germany; ^2^ Department of Psychiatry and Psychotherapy, Charité Universitätsmedizin Berlin, Corporate Member of Freie Universität Berlin Humboldt‐Universität zu Berlin, and Berlin Institute of Health Berlin Germany; ^3^ NeuroCure Clinical Research Center, Charité ‐ Universitätsmedizin Berlin, Corporate Member of Freie Universität Berlin Humboldt‐Universität zu Berlin, and Berlin Institute of Health Berlin Germany; ^4^ Experimental and Clinical Research Center (ECRC) Max Delbrueck Center for Molecular Medicine and Charité—Universitätsmedizin Berlin Berlin Germany; ^5^ VersMed Zürich Switzerland; ^6^ Department of Neurology Icahn School of Medicine at Mount Sinai New York New York USA

**Keywords:** emotion processing, MRI, multiple sclerosis

## Abstract

**Background:**

There is evidence that the processing of emotional information (EP) is altered in subjects with multiple sclerosis (MS). In a previous study, we found flattened emotional experience inpatients with early MS (clinically isolated syndrome and early relapsing/remitting MS) during the perception of emotional visual stimuli. The neural underpinnings of this finding are widely unknown.

**Objective:**

To investigate EP‐related brain mechanisms in patients with early MS and healthy controls (HC).

**Methods:**

Sixteen patients without neuropsychological deficits and sixteen matched HCs were presented with pictures with negative, positive, or neutral content while performing functional magnetic resonance brain scanning. Participants rated the induced emotion regarding valence and arousal using nine‐level Likert scales. Group differences and similarities in image category and valence/arousal associated brain responses and functional connectivity were assessed using Bayesian repeated measures analyses of covariance.

**Results:**

Patients reported less intense emotional experience of negative and positive emotional pictures. When presented with negative pictures, (1) brain response (BR) amplitudes were found to be increased in the dorsolateral prefrontal cortex (DLPFC) and middle temporal regions, including the amygdala and (2) functional connectivity (FC) between right amygdala and orbito‐frontal, ventromedial frontal, and ventral temporal regions was increased in patients with MS.

**Conclusion:**

Our findings of increased FC and BR in DLPFC and amygdala in MS patients with flattened emotional experience point to a disease‐related adaptive upregulation of the EP network. The latter is interpreted as emotion regulation of heightened sensitivity of amygdala activity to negative emotional content via increased fronto‐temporal functional connectivity.

## Graphical abstract

This fMRI‐study investigated emotional processing in patients with early MS and in healthy controls. We found flattened emotional experience associated with increased functional connectivity and brain response in the DLPFC and amygdala in patients. This suggests a disease‐related adaptive upregulation of the emotion processing and regulation network in patients with early MS.

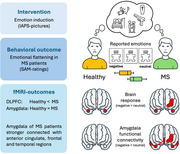

## 1 Introduction

Evidence suggests deficits in the processing of emotional information (EP) in patients with multiple sclerosis (MS). Reported EP deficits in MS include specific difficulties detecting particularly negative emotional facial expressions (A. Henry et al. 2017; J. D. Henry et al. 2009; Phillips et al. 2011), comprehending affective prosody (Adamaszek et al. 2022), recognizing emotional compared with neutral visual stimuli, or defective emotional enhancement of verbal memory (Grothe et al. 2021). In a previous study, we provided evidence that EP changes in MS do not seem to be secondary to other MS‐related neuropsychological or psychiatric symptoms and might be unmasked by a stressful environment even in patients with early MS (Hälbig et al. 2020). More specifically, patients with early MS rated the experience induced by visual emotional stimuli significantly less intense and less emotional than healthy controls (HCs).

The neural underpinnings of EP changes in MS are only partially known.Particularly, the neural substrate of reduced emotional experience in patients with early MS, as found in our previous study, has not been examined before. Impaired negative emotional facial recognition performance in moderately advanced MS was found to be correlated with decreased anterior insular and ventrolateral PFC activation in a functional magnetic resonance imaging (fMRI) study (Krause et al. 2009; Pfaff et al. 2019). In patients without EP deficits on emotional recognition tasks, enhanced brain activation in regions known to be involved in EP, such as the ventrolateral PFC, the posterior cingulate cortex (PCC) and precuneus, the occipital fusiform gyri and the anterior CC as well as a lack of functional connectivity (FC) between prefrontal areas and amygdalae, were found (Pfaff et al. 2019; Jehna et al. 2011, Passamonti et al. 2009). Interestingly, the role of the amygdala remains elusive since so far there is no evidence for EP‐related changes in amygdala BR in MS. Taken together, published data point to a disease‐related adaptation of emotion processing networks, including prefrontal areas and limbic structures such as the amygdala. Somewhat speculatively, these changes have been interpreted as a compensatory mechanism allowing for functional preservation of EP (or limiting the full expression of emotional symptoms) (Krause et al. 2009; Jehna et al. 2011; Passamonti et al. 2009).

The aim of this study was to ascertain fMRI brain responses (BR) and modulations in amygdala–PFC FC in patients experiencing visual emotional stimuli significantly less intense and less emotional than HCs (Hälbig et al. 2020). We used Bayesian statistics (see Glossary in Supporting Information) to overcome the limitations of frequentist statistical approaches which allowed testing not only for differences between HC and patients but also for similar activations of different areas (Wagenmakers 2007).

## 2 Materials and Methods

### 2.1 Participants

A total of 16 Caucasian right‐handed patients with clinically isolated syndrome (CIS) (*n* = 4, 2 female) or relapsing remitting MS (RRMS; *n* = 12; 6 female) meeting the diagnostic criteria for MS (Polman et al. 2011) within ≤ 5 years, and 16 right‐handed matched HCs (8 females) were tested (Table 1). The study was approved by the Charité—Universitätsmedizin Berlin ethics committee and registered at ClinicalTrials.gov (NCT02695394). Written informed consent was obtained from all participants. This sample was a subsample of the study reported by Hälbig and colleagues (Hälbig et al. 2020).

**TABLE 1:** Sample characteristics.

	RRMS/CIS	HC	Log_10_(BF10)
N^a^	16	16	−0.664
Female/Male^b^	8/8	8/8	−0.383
Age (years)^c^	32.63 (9.13)	28.44 (7.95)	−0.159
Education (years)^c^	15.57 (7.44)	17.78 (2.04)	−0.376
Diagnosis	12 RRMS; 4 CIS		
Disease duration (month)	29.19 (20.81)	N.A.	
Disability (EDSS)	1.18 (0.89)	N.A.	
Fatigue (MFIS)^c^	13.31 (12.97)	11.93 (9.48)	−0.450
Depression (HADS)^c^	1.75 (1.65)	1.63 (1.78)	−0.466
Depression (BDI‐2)^c^	4.38 (4.22)	3.25 (3.80)	−0.369
Anxiety (HADS)^c^	3.81 (2.37)	4.38 (1.75)	−0.376
Cognition (MMSE)^c^	29.93 (0.26)	29.69 (0.60)	−0.121
Cognition (BRB‐N)^c^			
SRT—LTS —CLTR —DR SPART—Learning —DR SDMT PASAT WLG	63.50 (9.30) 60.50 (12.01) 11.25 (1.57) 25.81 (3.31) 8.69 (2.24) 66.38 (14.55) 52.19 (6.59) 29.63 (5.90)	57.69 (10.22) 54.00 (14.23) 10.87 (1.45) 25.00 (3.50) 9.19 (1.28) 59.13 (9.88) 48.44 (9.17) 29.13 (5.28)	−0.012 −0.153 −0.391 −0.398 −0.374 −0.030 −0.183 −0.462
Quality of Life (SF‐36)^c^			
PCS—vitality —physical functioning —bodily pain —gen. health perc. MCS—physical role function —emotional role function —social role function —mental health	59.69 (18.30) 97.50 (3.61) 88.38 (19.42) 71.44 (12.98) 89.06 (20.35) 91.67 (22.77) 94.53 (7.86) 76.50 (10.82)	67.67 (10.68) 97.67 (5.30) 88.00 (19.13) 83.27 (13.02) 90.00 (22.76) 88.89 (20.57) 97.50 (7.01) 78.93 (11.16)	−0.116 −0.466 −0.467 **0.534** ^d^ −0.466 −0.449 −0.267 −0.404
Life satisfaction (SWLS)^c^	28.40 (4.00)	27.73 (2.99)	−0.419
Affective state (PANAS)^c^			
‐ positive ‐ negative	31.53 (5.00) 12.07 (3.17)	34.12 (7.57) 13.19 (3.56)	−0.263 −0.328

Abbreviations: BDI‐2, Beck Depression Inventory; BRB‐N, Repeatable Battery of Neuropsychological Tests; CIS, Clinically Isolated Syndrome; CLTR, consistent long‐term retrieval; DR, delayed recall; EDSS, Expanded Disability Status Scale; HADS, Hospital Anxiety and Depression Scale; HC, healthy control group; LTR, long‐term storage; MCS, Mental Health Score; MFIS, Modified Fatigue Impact Scale; MMSE, Mini‐Mental State Examination; PANAS, Positive and Negative Affect Scale; PASAT, Paced Auditory Serial Addition Test; PCS, Physical Health Score; RRMS, relapsing‐remitting MS; SDMT, Symbol Digit Modality Test; SF36, Short Form (36 item) Health Survey; SPART, 10/36—Spatial Recall Test; SRT, Selective Reminding Test; SWLF, Satisfaction with Life Scale; WLG, Word List Generation.

^a^ Bayesian Binomial Test.

^b^ Bayesian Contingency Table.

^c^ Bayesian independent samples *t*‐test.

^d^ moderate evidence for H1.

Exclusion criteria were cognitive deficits (Mini‐Mental State Examination [MMSE] Score < 25/30), clinically acute diseases within 7 days prior to the study evaluations, physical disabilities interfering with the study procedures, depression and anxiety (Hospital Anxiety and Depression Scale Score, HADS‐D > 7 and HADS‐A > 7, respectively) (Zigmond and Snaith 1983).

### 2.2 Neuropsychological Background Testing, Health‐Related Quality of Life, Satisfaction With Life, and Emotional Well‐being

To test verbal learning and memory, visuospatial learning and recall, attention, sustained attention, speed of information processing, and verbal fluency, the Brief Repeatable Battery of Neuropsychological Tests (BRB‐N) (Boringa et al. 2001) was used (Table 1 and Supporting Information). To evaluate for fatigue, the Modified Fatigue Impact Scale (MFIS) (Fisk et al. 1994) was used, and to probe for depression, the Beck Depression Inventory (BDI‐2) (Beck et al. 1994) was employed. Further, health‐related quality of life (SF‐36) (Morfeld et al. 2012), Satisfaction with life (Satisfaction with Life Scale, SWLS) (Diener et al. 1985), and emotional well‐being (Positive and Negative Affect Schedule, PANAS) (Watson et al. 1988) were examined.

### 2.3 Emotional Processing Testing

**FIGURE 1:**
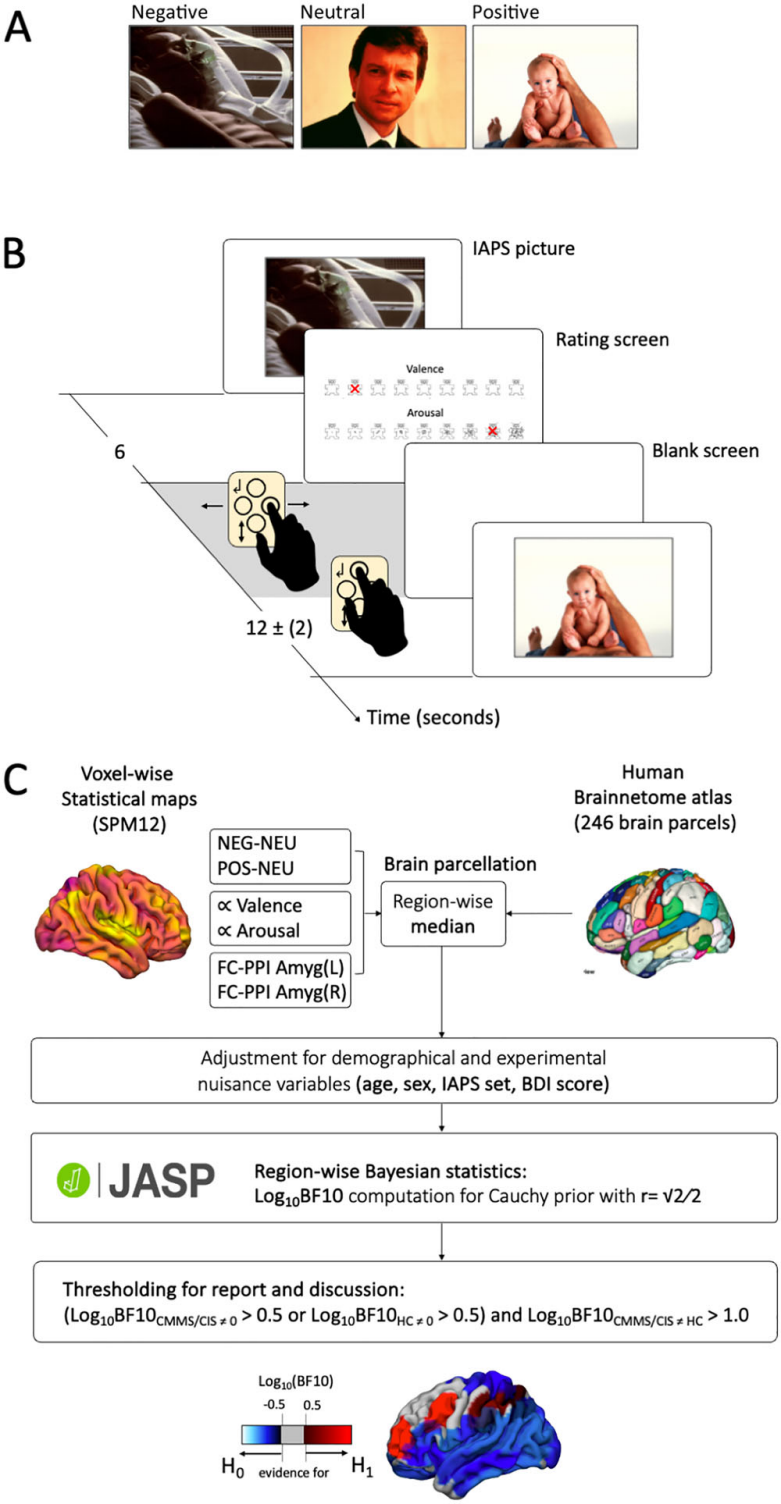
Exemplary stimuli for the three emotional valence categories (A) and 9‐level Self‐Assessment Manikin (SAM) Likert‐scales for the rating of affective picture components valence and arousal (B). Scheme depicting one trial of picture presentation and rating (C). Image analysis pipeline on group level (D).

Participants were presented with a series of 54 neutral and emotional pictures (e.g., accident victims, erotica, daily life objects, etc.) from the International Affective Picture System (IAPS) (Bradley and Lang 2007). These pictures differed in valence (18 positive, 18 neutral, 18 negative) and arousal (27 low, 27 high) based on standardized published rating values (Bradley and Lang 2007). Half of the pictures of each of the three valence categories had high and low arousal values, respectively (see Table S1) (Bradley and Lang 2007). Each picture was presented for 6 s. During this period, participants were instructed to focus on the picture and process its content. This was followed by a variable rating period of up to 8 s, during which participants rated the emotion elicited by the picture in terms of its valence and arousal using nine‐level Self‐Assessment Manikin Likert scales (Bradley and Lang 1994). Subsequently, a blank screen was shown for a flexible duration, ensuring an inter‐stimulus interval of 10 to 14 s (see Figure 1A,B). The selection of the timing parameters was chosen in accordance with previous research to allow proper emotion induction while avoiding carry over effects (Pfaff et al. 2019; Garrett and Maddock 2001).

After a delay of 15 min, there was a speeded recognition test for the stimuli previously presented (“old”) and for distractors (“new”), also comprising neutral and emotional pictures (Supporting Information). Experiential rating means, %‐correct recognized emotional items and the modulation of response time by emotional stimulus characteristics served as outcome variables (Hälbig et al. 2011).

### 2.4 Brain Scanning

Brain imaging was carried out with a three‐Tesla Trio TIM‐System (SIEMENS Healthcare GmbH, Erlangen) equipped with a 12‐channel phased‐array head coil at the Berlin Center for Advanced Neuroimaging (BCAN, Charité, Universitätsmedizin Berlin).

A total of 470 whole head functional brain images were acquired using a T2*‐weighted gradient echo planar imaging sequence (33 axial oriented slices, TR = 2000 ms, TE = 30 ms, flip angle = 78°, voxel size = 3 × 3 × 3 mm^3^). To model and correct nonlinear image distortions caused by field inhomogeneities and susceptibility artifacts, a gradient‐echo B0 field map (TR = 460, TE = 5.19/7.62 ms) was collected before functional imaging with the same spatial orientation and resolution as the functional images.

For anatomical reference and optimal warping of functional brain images into stereotactic standard space, a bias‐field corrected magnetization prepared double rapid‐acquisition gradient‐echo sequence with adiabatic inversion recovery (MP2RAGE) (McRae et al. 2010) were measured (TR = 5000 ms, TE = 2.91 ms, Times to inversion (TI) = 700/2500 ms, isometric voxel size = 1 × 1 × 1 mm^3^).

### 2.5 Processing of Brain Images

Data analysis was conducted using Statistical Parametric Mapping (SPM12, Wellcome Department of Imaging Neuroscience, London, UK, http://www.fil.ion.ucl.ac.uk/spm/).

Before submitting images to the SPM‐pipeline, all images were manually inspected for strong artifacts, reoriented to roughly match the orientation of the brain template of the International Consortium for Brain Mapping (ICBM; http://www.loni.ucla.edu/ICBM/), and the origin was set to the anterior commissure. Functional images were then corrected for acquisition delay, head motion, and nonlinear image distortions. A mean image was computed from artifact‐corrected functional time series. The structural MP2RAGE image was then co‐registered on this mean image, segmented into tissue classes, and warped into ICBM‐space using the unified segmentation algorithm described by Ashburner and Friston (2005). Using the linear and nonlinear warping parameters estimated during the segmentation process, also preprocessed functional images were transformed into ICBM‐space, resampled (voxel size = 3 × 3 × 3 mm^3^), and spatially smoothed using an isotropic Gaussian kernel (full width at half maximum, FWHM = 8 mm).

### 2.6 Modeling of Individual BR and FC

Most psycho–physiological EP studies evaluate the effects of standardized emotional stimuli on a psychophysiological parameter. These stimuli are usually defined by valence and arousal values as rated by standard reference populations. It is, however, well known that the emotional experience induced by a given stimulus varies considerably between individual subjects (Hoemann et al. 2020). To account for this source of variance, in the present study, we not only ascertained the BR as a function of standardized emotional image category but also the BR as a function of individual reported emotional valence and arousal of each picture.

To simplify the modelling and minimize collinearity among regressors (Mumford et al. 2015), we used three distinct models for each participant.

*Model 1*: BR (see Glossary in Supporting Information) during picture presentation was modeled for each emotional category separately by means of boxcar functions with uniform amplitudes (three regressors).

*Model 2*: To assess brain–behavior correlations, BR during picture presentation was modeled for valence and arousal separately by means of boxcar functions with amplitudes according to the SAM ratings (two regressors, parametrically modulated). Additionally, the mean response during image perception was modeled by a third regressor of boxcar functions with uniform amplitudes (one regressor).

*Model 3*: Differences in FC (see Glossary in Supporting Information) between amygdalae and distant brain regions as a function of image category were modeled using the psycho–physiological interaction approach (PPI, see Glossary in Supporting Information) (Friston et al. 1997; Gitelman et al. 2003). Seeds were left and right amygdalae as defined in Human Brainnetome atlas (https://atlas.brainnetome.org) (Fan et al. 2016). First eigenvariate time series were extracted from these seeds and two PPI regressors (NEG‐NEU; POS‐NEU) were created according to Gitelman et al. (Gitelman et al. 2003). The seed time series and the resulting PPI regressors were included in the model (three regressors), along with the task regressors (three regressors). PPI analyses were computed for left and right amygdala separately.

For all models, neural activities associated with button presses were modeled by means of one stick function per button press (one regressor). All BOLD predictors were obtained by convolving these neural models with the canonical hemodynamic response function (cHRF) (Friston et al. 1995). To account for signal fluctuations due to susceptibility × motion interactions, the movement parameters estimated during the motion correction step were considered as additional regressors of no interest (six regressors). Finally, all models include a constant to model allover signal mean.

Before fitting the model to the data, low‐frequency signal drifts in voxel time series were eliminated using a high‐pass filter with a cutoff frequency of 1/128 Hz. Physiological noise due to respiratory or aliased cardiologic effects was removed by means of autoregressive modeling. Model parameter weights were then estimated by restricted maximum likelihood (ReML) fit. In the next step, linear contrast images were computed for the conditions of interest for each subject.

### 2.7 Brain Parcellation and Parameter Extraction

Contrast images containing local estimates for (1) category‐specific BR (BR_NEG_ > BR_NEU_, BR_POS_ > BR_NEU_), (2) valence/arousal‐associated variation in BR (parametrically modulated BR), and (3) EP‐associated modulation in FC (PPI) were parcellated using the segmentation scheme of the Human Brainnetome atlas (see Figure 1). For each of the 32 participants, we extracted the median parameter estimates in the 246 atlas regions. The resulting 32 × 246 parameter vectors were adjusted for sex, age, and depression severity (BDI‐2 scores).

### 2.8 Statistical Analysis on Group Level

Statistical analyses were carried out by means of Bayesian methods (see Glossary in Supporting Information) with the software package JASP (https://jasp‐stats.org/) (JASP Team 2024). For all analyses, we used weakly informed Cauchy priors (*r* = √2⁄2, *t*‐tests, rmANCOVAs) (Ly et al. 2016; Rouder et al. 2012). After Monte Carlo Markov Chain (MCMC, with 5000 passes) simulation‐based estimation of posterior distributions, the common logarithm (Log_10_) of Bayesian factors for H1 (BF10, see Glossary in Supporting Information) was computed. In case of a positive Log_10_(BF10), data favors H1. In case of a negative one, data points to equality between the compared samples and favors H0.

For an absolute value of Log_10_(BF10) above 0.5, the favored hypothesis is three times more probable than its alternative, and the evidence strength is considered as moderate. In case of an absolute value of 1.0 and higher, the favored hypothesis is at least ten times more probable and the evidence for this hypothesis is strong (Jeffreys 1998; Kass and Raftery 1995). For a depiction of this pipeline, see Figure 1C.

#### 2.8.1 Demographic and Clinical Variables

Outcomes were analyzed descriptively, and differences between subgroups were ascertained by Bayesian binomial tests (prior) and independent sample *t*‐tests.

#### 2.8.2 Behavioral Endpoints

Statistical analyses of behavioral outcomes were also carried out by means of Bayesian methods. Mean valence and arousal ratings, mean reaction times during recognition, and recognition performance were analyzed descriptively and subjected to separate 2‐way Bayesian repeated‐measures Analyses of covariance with between‐subject factor GROUP (Patients, HC) and within‐subject factors image category (valence: negative, positive, neutral; arousal: high, low). To address potential paradigm unspecific effects on emotional processes, we used age, sex, IAPS‐set, and depression severity (BDI‐2) as covariates for the analyses of valence and arousal ratings, recognition performance, and response times. Models of interest consisted of single main effects of between or within‐subject factor of group (Model 1) or the main effect of group and the interaction valence/arousal*group (Model 2). To assess the evidence for an influence of these predictors on the data, these models were compared with the null model consisting of the allover intercept and the covariates of no interest, age, sex, IAPS‐set, BDI‐2, and subject and the inclusion Bayes factor logs Log_10_(BF_incl_) were computed via Bayesian model averaging. BF_incl_ quantifies the change from prior inclusion odds to posterior inclusion odds and can be interpreted as the evidence in the data for including a predictor. For example, a Log_10_(BF_incl_) of 1 implies that the data are 10 times more likely under the models that include the corresponding predictor than under the models without this predictor (Van Doorn et al. 2021).

Correlations between valence and arousal ratings corrected for age, sex, IAPS‐set, and depression severity (BDI‐2), and health‐related quality of life, satisfaction with life, emotional well‐being and fatigue were analyzed by means of Bayesian correlation analysis.

#### 2.8.3 Brain Imaging Endpoints

A brain region was considered for report and discussion only in case

(1) groups differ strongly (Log_10_(BF10) > 1.0) for the effect of interest in region‐wise two‐sided Bayesian independent sample *t*‐tests between the functional contrasts (e.g., ΔBR_N_ = BR_NEG−_BR_NEU_), and
(2) showed in at least one group at least moderate evidence for a main effect in post hoc one‐sided Bayesian one‐sample *t*‐tests for the group‐wise main effects (e.g., BR_NEG_).

Log_10_(BF10)_CMMS/CIS ≠ 0 _> 0.5 or Log_10_(BF10)_HC ≠ 0 _> 0.5) and Log_10_(BF10)_CMMS/CIS ≠ HC_ > 1.0

With this approach we aimed to exclude brain regions showing differences between groups without a sufficient main effect. In the interest of a more complete image, in figures we show all regions with at least moderate evidence for group differences.

## 3 Results

### 3.1 Behavioral Endpoints

**FIGURE 2:**
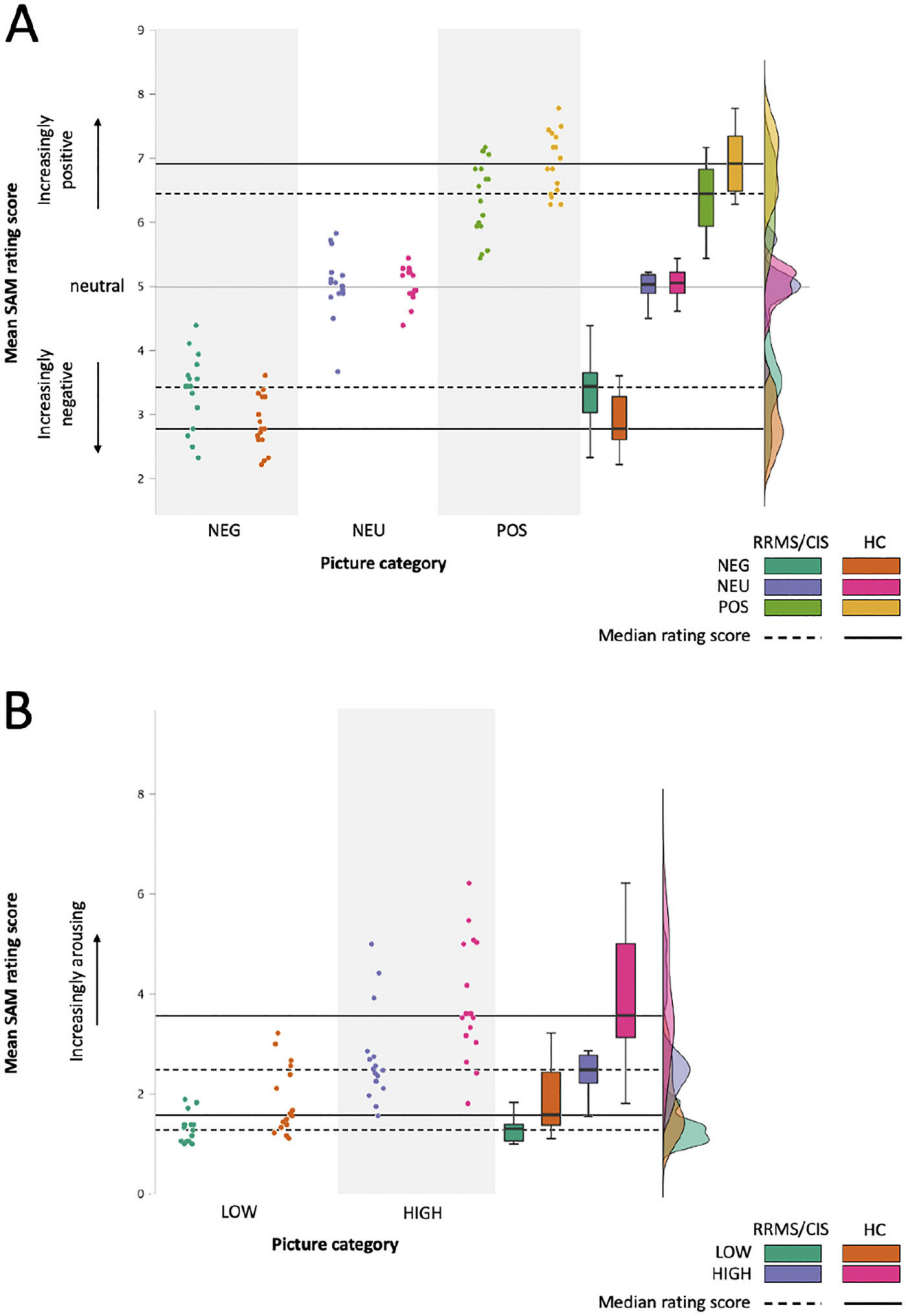
Emotional experience. IAPS‐picture ratings for the different emotional Valence (A) and Arousal (B) categories. Valence: NEG, negative; NEU, neutral; POS, positive. Arousal: LOW, HIGH.

There was strong evidence for group differences in valence and arousal ratings as indicated by the inclusion Bayesian factor for the interaction term of the compared models (Log_10_(BF_incl_) valence = 2.91; arousal = 1.12). Patients rated negative and positive IAPS‐pictures more neutral and less arousing than controls. In contrast, recognition performance and speed did not differ between groups. Evidence for this finding was moderate for response times (Log_10_(BF_incl_) = −0.74) and strong for performance (Log_10_(BF_incl_) = −1.6). See also Figure 2 and Table 2.

**TABLE 2:** Behavior: Bayesian repeated measures ANCOVA. Results of Bayesian model averaging.

	Predictor(s) average Log_10_(BF_incl_)			Post hoc	
Outcome	Image category	Group	Interaction	H+	Log_10_(BF+0)
Perception: valence rating	15.00	2.21	2.91	NEG: RRMS/CIS>HC POS: HC>RRMS/CIS	1.11 1.24
Perception: arousal rating	14.84	0.95	1.12	HIGH: HC>RRMS/CIS LOW: HC>RRMS/CIS	1.16 1.20
Recognition: performance	−1.08	−0.68	−1.60	n.a.	
Recognition: response time	−0.10	−0.31	−0.74	n.a.	

*Note*: All models include age, sex, IAPS‐set, BDI‐2 and subject.

Abbreviations: BF+0, Bayes factor for the comparison of H+ to H0; BFincl, Inclusion Bayes factor; brmANCOVA, Bayesian repeated measures analysis of covariance; n.a., not applicable.

### 3.2 Brain Imaging Endpoints

#### 3.2.1 Picture Category‐Associated BR (Model 1)

**FIGURE 3:**
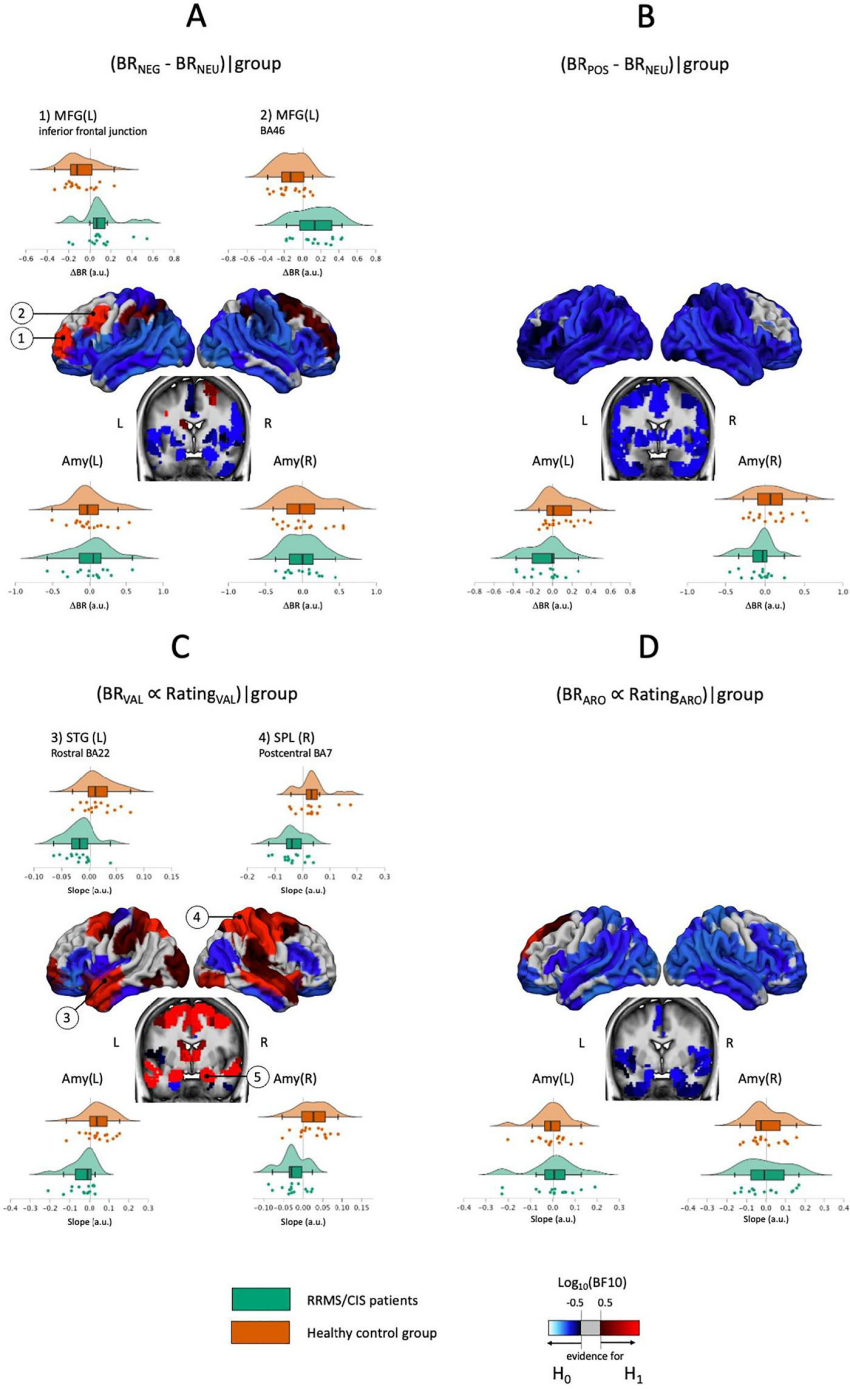
Picture content associated group differences and similarities in paradigm‐associated brain responses and functional connectivity during the encoding of negative compared to neutral IAPS pictures. (A) Mean brain responses (Model 1). (B) Rating correlated BR amplitudes (Model 2). Brain regions showing evidence for differences (H1 preferred) are shaded in red, those showing similarities between groups (H0 preferred) are shaded in blue. Brain areas in which the BOLD data support neither H0 nor H1 are shown in gray.

Δ*BR_N_ = BR_NEG−_BR_NEU_*: We found strong evidence for group differences in BR in the left Middle Frontal Gyrus in BA46 (Log_10_(BF10) = 1.70) and in the inferior frontal junction (ΔBR_N|RRMS/CIS_ ≠ ΔBR_N|HC_: Log_10_(BF10) = 1.04) (Table 3, Figure 3A).

**TABLE 3:** ΔBRN = BR_NEG−_BR_NEU_. Group differences in brain response to a picture with negative content.

					**Log_10_(BF10)** **(mean** ΔBR_N|*_ ± SD)		
		**Center (MNI)**			**Group difference** ^a^	ΔBR_N|*_ > 0 | ΔBR_N|*_ < 0 ^b^	
**Brain region (functional area)**	**H**	***x***	***y***	***z***		**RRMS/CIS**	**HC**
Middle frontal gyrus (BA46, DLPFC)	L	−28	56	12	**1.70**	**0.660 | −1.027** (0.133** ± **0.213)	**−1.102 | 1.249** (−0.126** ± **0.156)
Middle frontal gyrus (inferior frontal junction)	L	−42	13	36	**1.04**	**0.441 | −0.996** (0.101** ± **0.186)	**−1.041 | 0.661** (−0.089** ± **0.147)
Amygdala (medial)	L	−19	−2	−20	**−0.512**	**−0.536 | −0.623** (0.010** ± **0.288)	**−0.613 | −0.573** (−0.004** ± **0.260)
Amygdala (medial)	R	19	−2	−19	**−0.505**	**−0.607 | −0.554** (−0.005** ± **0.227)	**−0.508 | −0.668** (0.019** ± **0.305)

Abbreviations: BA, Brodmann area; CIS, Clinically Isolated Syndrome; DLPFC, dorsolateral prefrontal cortex; HC, healthy control group; H, hemisphere; MNI, Montreal Neurological Institute standard space; RRMS, relapsing‐remitting MS.

^a^ Two‐sided Bayesian independent sample *t*‐test (ΔBR_N|RRMS/CIS_ ≠ ΔBR_N|HC_).

^b^ One‐sided Bayesian one sample *t*‐test (ΔBR_N|*_ > 0 | ΔBR_N|*_ < 0).

In BA46, RRMS/CIS patients showed moderate evidence for an increase in BR during the presentation of negative compared to neutral picture content (ΔBR_N|RRMS/CIS_ > 0: Log_10_(BF10) = 0.660), whereas in HCs we found strong evidence for a decrease in BR (ΔBR_N|HC_ < 0: Log_10_(BF10) = 1.249) in this region. In the same vein, in the inferior frontal junction, HCs showed a, however, less pronounced, decrease in BR ((ΔBR_N|HC_ < 0: Log_10_(BF10) = 0.661).

Notably, there were no group differences in mean amygdala activity, but moderate evidence of their equality (Log_10_(BF10) > −0.5).

Δ*BR_P_ = BR_POS−_BR_NEU_*: There was neither a difference in BR during the perception of positive pictures versus neutral pictures nor between groups (Table 3, Figure 3B).

#### 3.2.2 Brain–Behaviour Correlation: Emotional Experience Associated BR (Model 2)

*Valence*: There was evidence for group differences in the correlation between perceived picture valence and BR amplitude in right premotor cortex (BA6: Log_10_(BF10) = 1.81), middle and inferior temporal regions (right MTG/left STG BA22: Log_10_(BF10) = 1.63), superior parietal lobule (SPL left and right: all Log_10_(BF10) > 1.076) and the amygdalae bilateral (Log_10_(BF10) > 1.033). RRMS/CIS patients showed in three of these parcels a strong negative correlation and in seven a moderate correlation, indicating the highest brain activity during the presentation of the most negative experienced pictures, whereas HCs showed in eleven regions moderate positive correlations (Table 4, Figure 3C).

**TABLE 4:** BR = *m* × Rating_VAL _+ *c*. Group differences in valence associated amplitude in brain response.

		Center (MNI)			Log_10_(BF10) (mean slope ± SD)		
Brain region (functional area)	H				Group difference^a^	*m* > 0 | *m* < 0^2^	
		*x*	*y*	*z*		RRMS/CIS	HC
Precentral gyrus (caudal BA 6)	R	33	−7	57	**1.810**	**−1.006 | 1.010** (−0.028** ± **0.050)	**0.887 | −1.071** (0.027** ± **0.42)
Superior temporal gyrus (rostral BA22)	L	−55	−3	−10	**1.631**	**−1.071 | 1.088** (−0.018** ± **0.025)	**0.676 | −1.046** (0.016** ± **0.028)
Middle temporal gyrus	R	58	−16	−10	**1.628**	**−1.071 | 1.087** (−0.023** ± **0.041)	**0.687 | −1.046** (0.022** ± **0.038)
Superior parietal lobule (intraparietal BA7)	L	−16	−60	63	**1.386**	**−1.056 | 0.894** (−0.041** ± **0.071)	**0.570 | −1.032** (0.042** ± **0.073)
Superior parietal lobule (caudal BA7)	L	−15	−71	52	**1.221**	**−1.060 | 0.958** (−0.049** ± **0.082)	**0.421 | −1.004** (0.049** ± **0.090)
Superior parietal lobule (intraparietal BA7)	R	19	−57	65	**1.223**	**−1.000 | 0.474** (−0.038** ± **0.051)	**0.820 | −1.060** (0.034** ± **0.066)
Superior parietal lobule (lateral BA5)	R	23	−43	67	**1.159**	**−1.000 | 0.480** (−0.031** ± **0.050)	**0.748 | −1.056** (0.027** ± **0.050)
Inferior parietal lobule (rostro‐dorsal BA40)	R	47	−35	45	**1.076**	**−1.004 | 0.495** (−0.024** ± **0.044)	**0.616 | −1.036** (0.026** ± **0.039)
Postcentral gyrus (BA2)	R	48	−24	48	**1.069**	**−1.009 | 0.533** (−0.032** ± **0.042)	**0.566 | −1.027** (0.030** ± **0.044)
Amygdala (medial)	L	−19	−2	−20	**1.066**	**−1.000 | 0.477** (−0.038** ± **0.068)	**0.624 | −1.036** (0.042** ± **0.072)
Amygdala (lateral)	R	28	−3	−20	**1.033**	**−1.018 | 0.583** (−0.025** ± **0.032)	**0.487 | −1.018** (0.024** ± **0.039)

Abbreviations: BA, Brodmann area; BR, brain response; CIS: Clinically Isolated Syndrome; HC, healthy control group; H, hemisphere; MNI, Montreal Neurological Institute standard space; RRMS, relapsing‐remitting MS; VAL, Valence.

^a^ Two‐sided Bayesian independent sample *t*‐test (*m* ≠ 0).

^b^ One‐sided Bayesian one sample *t*‐test (*m* > 0 | *m* < 0).

*Arousal*: There was no evidence for an association between subjects’ reports of experienced picture arousal and BR (Table 4, Figure 3D).

#### 3.2.3 Stimulus Dependent Differences in Amygdala FC (Model 3)

**FIGURE 4:**
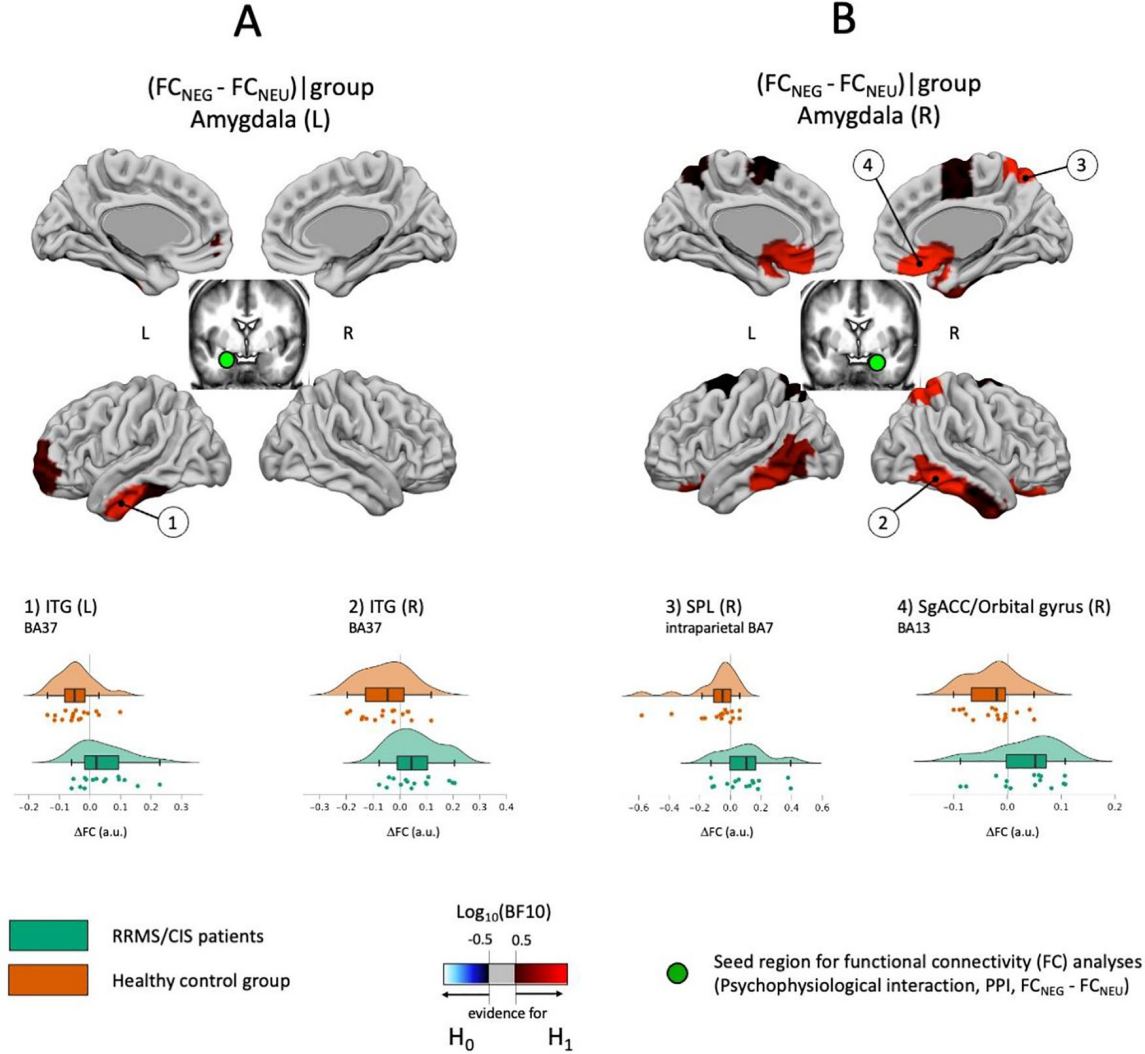
Picture content associated group differences and similarities in functional connectivity (Model 3) during the encoding of negative compared to neutral IAPS pictures. (A) Modulation in functional connectivity (PPI) with the left Amygdala. (B) Modulation in functional connectivity (PPI) with the right Amygdala. Seed regions are marked with green circles. Brain regions showing evidence for differences (H1 preferred) are shaded in red, those showing similarities between groups (H0 preferred) are shaded in blue. Brain areas in which the BOLD data support neither H0 nor H1 are shown in gray.

*Right Amygdala*: During the presentation of negative pictures, we found group differences in FC between right Amygdala and right inferior temporal (ITG, BA37, Log_10_BF10 = 1.26), right superior parietal (SPL, BA7, Log_10_BF10 = 1.08) and right ventral frontal structures (Orbital Gyrus, subgenual Anterior Cingulate Cortex, Log_10_BF10 = 1.02). In MS patients a moderate increase of FC with ITG and SPL was found. In HC, FC decreased moderately along all connections (Table 5, Figure 4A,B).

**TABLE 5:** ΔFC = FC_NEG−_FC_NEU_. Group differences in emotion‐dependent functional connectivity between Amygdalae and distant brain regions (PPI). ΔFC = FC_NEG−_FC_NEU._

		Center (MNI)			Log_10_BF10 (mean ΔFC ± SD)					
					Group difference^a^		ΔFC > 0 | ΔFC < 0^b^			
							RRMS/ CIS	HC	RRMS/ CIS	HC
					Seed Amygdala					
**Brain region** (functional area)	**H**	***x***	***y***	***z***	**L**	**R**	**L**		**R**	
**Inferior Temporal Gyrus** (BA37)	**R**	61	−40	−17		**1.26**			**0.666 | −0.991** (0.057** ± **0.091)	**−1.036 | 0.632** (−0.053** ± **0.090)
**Superior Parietal Lobule** (intraparietal BA7)	**R**	19	−57	65		**1.08**			**0.554 | −1.538** (0.093** ± **0.158)	**−1.027 | 0.561** (−0.096** ± **0.169)
**Orbital Gyrus/sgACC** (BA13, OFC)	**R**	9	20	−19		**1.02**			**0.433 | −0.991** (0.033** ± **0.062)	**−1.041 | 0.665** (−0.027** ± **0.045)
**Inferior Temporal Gyrus** (BA37)	**L**	−51	−57	−15	**1.16**		**0.338 | −0.975** (0.041** ± **0.082)	**−1.081 | 1.003** (−0.044** ± **0.061)		

Abbreviations: BA, Brodmann area; CIS, Clinically Isolated Syndrome; H, hemisphere; HC, healthy control group; MNI, Montreal Neurological Institute standard space; OFC, orbitofrontal cortex; RRMS, relapsing‐remitting MS; sgACC, subgenual anterior cingulate cortex.

^a^ Two‐sided Bayesian independent sample *t*‐test (ΔFC ≠ 0).

^b^ One‐sided Bayesian one sample *t*‐test (ΔFC > 0 | ΔFC < 0).

*Left amygdala*: The group differences in FC with distant brain regions were limited to left inferior temporal regions (ITG, BA37, Log_10_BF10 = 1.16), with strong evidence for a decrease in the HC group.

Notably, no evidence for interhemispheric modulations in amygdala FC was found.

## 4 Discussion

There is converging evidence that the processing of emotional content may be altered in patients with MS. In our previous study, patients with early MS without cognitive deficits experienced emotional visual stimuli less intensely than matched HCs (Hälbig et al. 2020) when tested in a stressful environment. Here, we confirm these findings and report neural underpinnings.

### 4.1 Methodological Differences to Previous EP‐Studies in MS

Psychophysiological research usually uses frequentist statistics. To overcome the limitations of frequentist statistical approaches, our study employed Bayesian statistics, which allowed testing not only for differences between HC and patients and differences between BR of different areas, but also for similarities. Further, the BR to emotional stimuli was ascertained as a function of individual‐reported emotional valence and arousal of each stimulus. This approach allowed us to eliminate the bias of analyses based solely on standard emotional categories.

### 4.2 Behavioral Results and Their Relationship to Previous Findings

Behavioral findings analyzed with Bayesian statistics fully confirm the results of the frequentist analysis as reported in our previous paper and confirm that flattened experience of emotional visual stimuli in MS may be present even in early MS and are not restricted to negative information but may affect the full range of emotional valence as well as the arousal dimension (Hälbig et al. 2020). Further, EP changes in early MS do not seem to be secondary to other MS‐related neuropsychological or psychiatric symptoms.

### 4.3 Flattened Emotional Experience of Negative Stimuli Is Associated With Increased BR in DLPFC and Amygdala and Increased FC Between these Structures

MS patients in the present study exhibited increased BR‐amplitudes *in the dorsolateral prefrontal cortex, the ventral prefrontal cortex, the temporal pole, and the SPL* when presented with negative emotional pictures. Increases of BR consistently were found to be induced by both, standard negative emotional stimuli (Model 1) and stimuli that were experienced as negative by the respective individual (Model 2).

Altered BR‐amplitudes in prefrontal areas during EP‐tasks have been previously reported by fMRI studies using types of emotional stimuli differing considerably from the visual IAPS stimuli in the present study (Krause et al. 2009; A. Henry et al. 2017). On an emotional facial expression matching‐to‐sample task, enhanced BR was found in *ventrolateral PFC*, the *PCC* and *precuneus*, the *occipital fusiform gyri*, and the *anterior CC* (Passamonti et al. 2009) in patients with early MS without EP‐deficits. Interestingly, in patients with moderately advanced negative impaired emotional facial recognition performance was associated with decreased *anterior insular* and *ventrolateral PFC* BR (Krause et al. 2009). The heterogeneity of these findings is likely due to methodological differences and differences in the populations studied. Nevertheless, there is converging evidence for altered PFC activation.

Building on previous research, our study found that the trial‐by‐trial amplitude of BR during the processing of negative IAPS pictures was negatively correlated with valence in limbic structures, but only in patients. These structures include the *superior parietal lobule, inferior temporal regions, and, most notably, the amygdala, bilaterally*. EP‐related changes in amygdala BR have not been reported previously in MS. It remains to be determined whether discrepancies with earlier studies (Krause et al. 2009, Passamonti et al. 2009) arise from differences in experimental design or trial‐by‐trial data modelling based on patients’ valence ratings (Model 2).

Finally, FC between *right amygdala* and *orbito‐frontal*, *ventro‐medial frontal*, and *ventral temporal regions* during presentation of negative pictures was increased in MS patients. Amygdala–PFC connectivity changes have been reported related to EP in MS (Passamonti et al. 2009). Previous research reported *decreased* FC, however, using an emotional facial expression task. Irrespective of the methodological task differences, results of the present study confirm altered FC of the DLPFC‐amygdala pathways in emotional processing in MS.

### 4.4 Interpretation of Behavioral Findings and Pattern of BR

The present findings of emotional flattening and increased BR in PFC and amygdalae, known as key regions mediating EP, in conjunction with altered FC of these structures, confirm the notion of MS‐related functional changes of the emotional processing network (Krause et al. 2009; Braunstein et al. 2017; Berboth and Morawetz 2021).

Currently, no data‐driven conclusions regarding the underlying mechanisms can be drawn. However, BR changes and altered connectivity have been interpreted as a compensatory mechanism allowing to functionally preserve EP (or limit the full expression of emotional symptoms) in patients with MS (Krause et al. 2009; Jehna et al. 2011; Passamonti et al. 2009). This mechanism is thought to involve the reallocation of neural resources in specific brain areas adjacent or functionally‐related to dysfunctional processes, potentially through the employment of neurocognitive strategies (Kuhlmann et al. 2023; Chard et al. 2021). Nevertheless, this interpretation should be taken with caution, especially in the absence of behavioral deficits or acute symptoms.

We propose considering an alternative, yet speculative, framework that also accounts for the potential role of the amygdala (Anders et al. 2004). Amygdala responses were strongly correlated with stimulus valence in our patients (Fig 3C), with the highest brain activity during the presentation of the most negative pictures. Based on this finding, and seemingly in contrast to the flattened emotional experience in our patients, it is tempting to speculate that MS patients are basically *more* susceptible to emotionally valent information than HCs. Increased susceptibility to negative content is a well‐known phenomenon present in patients with substantial chronic disease (Orzechowska et al. 2023) and has been shown to prompt emotion regulatory processes aiming at suppressing associated negative emotions to maintain mental well‐being. It is therefore conceivable that the perception of distressing content in our patients triggered emotion regulation associated with prefrontal top‐down processes (Braunstein et al. 2017; McRae et al. 2010; Ochsner and Gross 2008) via increased functional prefrontal cortex and amygdala connectivity, resulting, however, at the behavioral level in emotionally flattened experience.

## 5 Limitations

Our study has several limitations, which should be considered in future research. The study sample was relatively small, albeit homogeneous and well‐matched. Importantly, although BR and FC data do not point to structural changes of the EP relevant frontal and temporal structures, we did not investigate lesion load in our patients. Therefore, we are not able to conclude whether the frontobasal fiber connections, which are particularly important for the interpretation of our connectivity findings, were unaffected by the disease. Moreover, future studies should examine explicit and implicit emotion regulation to substantiate the claim that flattened emotional experience might result from an overshooting emotional regulation of heightened emotional sensitivity. Another relevant question that should be addressed in further research is the investigation of longitudinal changes in EP associated with different stages of the disease.

Finally, although neuropsychological background testing, health‐related quality of life, satisfaction with life and emotional well‐being in the present study do not point to a clinical relevance of flattened emotional experience in early stages of MS, the clinical relevance of a flattened emotional response as ascertained by experimental study designs remains to be clarified, especially in the context of the chronic course of the disease over the entire life span.

## 6 Conclusion

We extend current knowledge on EP in MS by demonstrating flattened emotional response without obvious neuropsychiatric disease early during MS. The neural correlates reported here point to a functional adaptation of the prefrontal–amygdala networks as the key structure implicated in EP.

## Author Contributions

**Torsten Wüstenberg**: conceptualization, methodology, software, data curation, formal analysis, visualization, writing – review and editing, writing – original draft, project administration, investigation. **René Gieß**: investigation, validation, writing – review and editing, project administration. **Judith Bellmann‐strobl**: conceptualization, methodology, supervision, writing – review and editing. **Hagen Kunte**: conceptualization, supervision, investigation, validation, writing – review and editing, project administration. **Friedemann Paul**: conceptualization, methodology, supervision, resources, project administration, funding acquisition, writing – original draft, writing – review and editing. **Thomas D. Hälbig**: conceptualization, methodology, data curation, formal analysis, supervision, funding acquisition, project administration, resources, writing – original draft, writing – review and editing.

## Peer Review

The peer review history for this article is available at https://publons.com/publon/10.1002/brb3.70987.

## Supporting information




**Supplementary Materials**: brb370987‐sup‐0001‐SuppMat.docx

## Data Availability

The data that support the findings of this study are available on request from the corresponding author. The data are not publicly available due to privacy or ethical restrictions.
