# Supplementary material for: Neural Signatures of Flattened Emotional Experience in Patients With Early Multiple Sclerosis: A Bayesian Approach
Source: Brain Behav. 2025 Oct 29;15(11):e70987. doi: 10.1002/brb3.70987 (PMC12571962; doi:10.1002/brb3.70987)
Supplement: Supplementary file 1 — Supplementary Materials: brb370987‐sup‐0001‐SuppMat.docx [file BRB3-15-e70987-s001.docx]

**Supplementary Material**

**Neural Signatures of Flattened Emotional Experience in Patients with Early Multiple Sclerosis: A Bayesian Approach**

Torsten Wüstenberg,^1,2^ René Gieß,^3^ Judith Bellmann-Strobl, ^3,4^ Hagen Kunte,^3,5^
Friedemann Paul^3^ & Thomas D. Hälbig^3,6^

^1^ Core Facility for Neuroscience of Self-Regulation (CNSR), Field of Focus 4 (FoF4), Heidelberg University, Heidelberg, Germany

^2^ Department of Psychiatry and Psychotherapy, Charité Universitätsmedizin Berlin, corporate member of Freie Universität Berlin, Humboldt-Universität zu Berlin, and Berlin Institute of Health, Berlin, Germany

^3^ NeuroCure Clinical Research Center, Charité - Universitätsmedizin Berlin, corporate member of Freie Universität Berlin, Humboldt-Universität zu Berlin, and Berlin Institute of Health, Berlin, Germany

^4^ Experimental and Clinical Research Center (ECRC), Max Delbrueck Center for Molecular Medicine and Charité – Universitätsmedizin Berlin, Germany

^5^ VersMed, Zürich, Switzerland

^6^ Department of Neurology, Icahn School of Medicine at Mount Sinai, New York, NY, USA

**Glossary (in alphabetical order)**

**Bayes factors (BF)** are statistical parameters used to quantify the evidence for one hypothesis over another, based on observed data. It is central to Bayesian hypothesis testing and offers an alternative to traditional p-values. The Bayes factor is the ratio of the 🡪 **likelihood** of the data under one hypothesis to the likelihood under another hypothesis, typically the alternative (H1) vs. the null hypothesis (H0). In this case, a BF greater than 1 indicates evidence in favor of the numerator hypothesis, while a value less than 1 favors the denominator. The type of ratio used is added to the abbreviation: BF10 = P(D|H1)/P(D|H0) or BF01 = P(D|H0)/P(D|H1). To overcome the non-linearity problem of ratios, the 🡪 **decadic logarithm** of the BF (Log_10_BFx) is often used for report. Unlike Frequentist statistics that relies on the concept of significance, BFs provide a graded measure of evidence, not just a binary decision. Therefore, the magnitude of the Bayes factor reflects the strength of evidence, with established interpretive scales (e.g., Jeffreys’ scale). Importantly, the interpretation depends on the choice of priors and the models compared.

**Bayesian statistics** is a branch of statistics that uses Bayesian inference, which is based on the idea of updating the probability for a hypothesis as more evidence or information becomes available (e.g. by experiments). It’s a different approach to statistical analysis than traditional frequentist methods. Bayesian inference starts with a *prior distribution* that reflects our initial knowledge about the available data. Newly collect data, which add new information about the “true” distribution are used to update the prior distribution using 🡪 **Bayes' theorem** to obtain the *posterior distribution*. Statistical inferences can be made by comparing the parameters of the prior and posterior distributions. 🡪 **Bayes factors (BFs)** are often used for reporting inference results.

**Bayes' theorem** is a mathematical formula that describes how to update the probability of a hypothesis based on new evidence. It's named after Thomas Bayes, an 18th-century English statistician who first formulated it. **P(H|D) = P(D|H) * P(H) / P(D).** Where P(H|D) is the posterior probability of hypothesis H given the data D**;** P(D|H) is the 🡪 **likelihood function**, representing the probability of observing D if H were true**;** P(H) is the prior probability of H before considering D**;** P(D) is the marginal 🡪 **likelihood** of D.

The term **brain response (BR)** refers to how the brain reacts to various internal or external stimuli, including sensory input, stress, injury, or cognitive demands. This response can be measured at multiple levels, from molecular and cellular changes to large-scale neural network activity. In functional magnetic resonance imaging (fMRI), the blood oxygenation level dependent (BOLD) effect is used, to detect the local increase in blood flow indicating the BR-associated neural activity. In an experimental context, BRs are provoked by the experimental paradigm. To detect the local BR, linear models are fitted to the data. These models usually contain regressors that model the mean BR for a specific stimulus category. To also quantify the proportion of BR associated with specific stimulus characteristics, these models can include 🡪 **parametrically modulated regressors**.

**Log_10_** or the decadic logarithm, is a mathematical function that maps a positive real number to its power of 10 (Examples: Log10(100) = 2 because 10^2^ = 100; Log_10_(0.1) = -1 because 10^-1^ = 0.1; Log_10_(1) = 0 because 10^0^ = 1). Using the decadic logarithm, the hyperbolic progression of the original Bayes factors is transformed into a linear function, making it easier to interpret, as the evidence strengths are linearly symmetrical to zero.

**Functional connectivity (FC)** analysis is a correlation-based method in functional MRI (fMRI) research. The base assumption of this analysis is, that brain regains with similar or even comparable signal time series are very likely involved in the same functional neural network and are therefore functionally connected. According to the extraction of the time series, one can distinguish between seed-, parcel- and voxel-based FC analyses. In seed-based FC analyses the time series from a certain seed region (e.g. amygdala) is correlated with the time series in all other voxels of the brain. In parcel-based FC analyses, brain atlases are used to extract mean time series within the compartments of the brain atlas (parcels). These time series were than correlated with each other, forming a network. Therefore, parcel-based FC computation is an important prerequisite for brain network analyses using graph theoretical approaches. In the less-common voxel-based FC analyses, cross correlations of the time series in each voxel were computed resulting in a huge number of FC probability map (one for each voxel).

**Likelihood** is a measure of how likely it is that a set of observations or data would occur given a particular model or hypothesis. Given a statistical model with parameters θ, the **likelihood function L(θ)** is defined as: L(θ) = P(X|θ) or even L(D) = P(D|H).

A **parametrically modulated regressor** is a predictor variable that is multiplied by a function of another predictor variable, usually in the form of a parameterization. This enables the model to adapt to the different levels or scales of the predictor variable. Example: To capture the effect of emotional valence on the intensity of the 🡪 **brain response (BR)**, the predictor modelling mean BR is multiplied by trial-wise valence ratings from a given participant and mean-adjusted. The resulting predictor shows a specific amplitude for each evoked brain response (one per trial in event-related settings like ours). Thus, this regressor is able to explain additional variance beyond the mean BR and identify areas with valence-correlated variations in BR.

**Psycho-physiological interaction (PPI)** analysis is a method in functional MRI (fMRI) research to investigate how the connectivity between brain regions changes depending on the experimental context. To this end, PPI analysis examines whether the relationship (functional connectivity) between a seed brain region and other regions is modulated by a specific psychological task or condition. In other words, it tests if the influence of one brain area on another varies according to the experimental context, providing insight into context-dependent changes in functional connectivity.

**Supplementary Table S1:** List of International Affective Picture System (IAPS) pictures used in our study. Category, picture number, description and normative valence and arousal values (Lang et al., 2008).

| Set A | |  |  |  | Set B | |  |  |  |
| --- | --- | --- | --- | --- | --- | --- | --- | --- | --- |
| PERCEPTION & RECOGNITION | | | | | | | | | |
| Category | # | Description | Valence | Arousal | Category | # | Description | Valence | Arousal |
| **Negative** | 1070 | Snake | 3.96 | 6.16 | **Negative** | 2230 | Sad Face | 4.53 | 4.13 |
|  | 1090 | Snake | 3.70 | 5.88 |  | 2312 | Mother | 3.71 | 4.02 |
|  | 1275 | Roaches | 3.30 | 4.81 |  | 2590 | Eld. Woman | 3.26 | 3.93 |
|  | 2520 | Elderly | 4.13 | 4.22 |  | 3063 | Mutilation | 1.49 | 6.35 |
|  | 2722 | Jail | 3.47 | 3.52 |  | 3068 | Mutilation | 1.80 | 6.77 |
|  | 3030 | Mutilation | 1.91 | 6.76 |  | 3102 | Burn. Victim | 1.40 | 6.58 |
|  | 3100 | Burn. Victim | 1.60 | 6.49 |  | 3170 | Baby | 1.46 | 7.21 |
|  | 3140 | Body | 1.83 | 6.36 |  | 3180 | Battered | 1.92 | 5.77 |
|  | 3150 | Finger | 2.26 | 6.55 |  | 3230 | Dying | 2.02 | 5.41 |
|  | 6010 | Jail | 3.73 | 3.95 |  | 3550 | Injury | 2.54 | 5.92 |
|  | 6200 | Aimed Gun | 2.71 | 6.21 |  | 7234 | Iron. Board | 4.23 | 2.96 |
|  | 6350 | Attack | 1.90 | 7.29 |  | 7700 | Office | 4.25 | 2.95 |
|  | 7060 | Trash Can | 4.43 | 2.55 |  | 9000 | Cemetery | 2.55 | 4.06 |
|  | 9010 | Barbed Wire | 3.94 | 4.14 |  | 9001 | Cemetery | 3.10 | 3.67 |
|  | 9110 | Puddle | 3.76 | 3.98 |  | 9140 | Cow | 2.19 | 5.38 |
|  | 9330 | Garbage | 2.89 | 4.35 |  | 9180 | Seal | 2.99 | 5.02 |
|  | 9331 | Homeless | 2.87 | 3.85 |  | 9250 | War Victim | 2.57 | 6.60 |
|  | 9390 | Dishes | 3.67 | 4.14 |  | 9265 | Hung Man | 2.60 | 4.34 |
|  |  |  |  |  |  |  |  |  |  |
| **Neutral** | 2200 | Neutral | 4.79 | 3.18 | **Neutral** | 2190 | Man | 4.83 | 2.41 |
|  | 2214 | Man | 5.01 | 3.46 |  | 5534 | Mushroom | 4.84 | 3.14 |
|  | 2381 | Girl | 5.25 | 3.04 |  | 5740 | Plant | 5.21 | 2.59 |
|  | 2890 | Twins | 4.95 | 2.95 |  | 7002 | Towel | 4.97 | 3.16 |
|  | 5500 | Mushroom | 5.42 | 3.00 |  | 7006 | Bowl | 4.88 | 2.33 |
|  | 5510 | Mushroom | 5.15 | 2.82 |  | 7031 | Shoes | 4.52 | 2.03 |
|  | 7004 | Spoon | 5.04 | 2.00 |  | 7035 | Mug | 4.98 | 2.66 |
|  | 7025 | Stool | 4.63 | 2.71 |  | 7090 | Book | 5.19 | 2.61 |
|  | 7034 | Hammer | 4.95 | 3.06 |  | 7130 | Truck | 4.77 | 3.35 |
|  | 7037 | Trains | 4.81 | 3.71 |  | 7185 | Abstract | 4.97 | 2.64 |
|  | 7041 | Baskets | 4.99 | 2.60 |  | 7190 | Clock | 5.55 | 3.84 |
|  | 7050 | Hair dryer | 4.93 | 2.75 |  | 7207 | Beads | 5.15 | 3.57 |
|  | 7150 | Umbrella | 4.72 | 2.61 |  | 7493 | Man | 5.35 | 3.39 |
|  | 7160 | Fabric | 5.02 | 3.07 |  | 7550 | Office | 5.27 | 3.95 |
|  | 7175 | Lamp | 4.87 | 1.72 |  | 7820 | Agate | 5.39 | 4.21 |
|  | 7182 | Checkerboard | 5.16 | 4.02 |  | 7950 | Tissue | 4.94 | 2.28 |
|  | 7184 | Abstract Art | 4.84 | 3.66 |  | 9070 | Boy | 5.01 | 3.63 |
|  | 7705 | Cabinet | 4.77 | 2.65 |  | 9700 | Trash | 4.77 | 3.21 |

| **Table S1** continued | | | | | | | | | |
| --- | --- | --- | --- | --- | --- | --- | --- | --- | --- |
|  |  |  |  |  |  |  |  |  |  |
| **Positive** | 1540 | Cat | 7.15 | 4.54 | **Positive** | 1600 | Horse | 7.37 | 4.05 |
|  | 1750 | Bunnies | 8.28 | 4.1 |  | 1610 | Rabbit | 7.69 | 3.98 |
|  | 2050 | Happy baby | 8.20 | 4.57 |  | 2030 | Woman | 6.71 | 4.54 |
|  | 2160 | Fath. & baby | 7.58 | 5.16 |  | 2040 | Happy baby | 8.17 | 4.64 |
|  | 2250 | Baby | 6.64 | 4.19 |  | 2057 | Baby & Fath. | 7.81 | 4.54 |
|  | 2340 | Older man | 8.03 | 4.9 |  | 2240 | Neutral | 6.53 | 3.75 |
|  | 4210 | Female Nude | 5.72 | 6.08 |  | 4250 | Erotic fem. | 6.79 | 5.16 |
|  | 4572 | Erotic Male | 6.15 | 4.8 |  | 4530 | Nude male | 6.19 | 5.31 |
|  | 4610 | Couple | 7.29 | 5.1 |  | 4624 | Couple | 6.84 | 5.02 |
|  | 4659 | Erotic Couple | 6.87 | 6.93 |  | 4680 | Erotic couple | 7.25 | 6.02 |
|  | 5200 | Flowers | 7.36 | 3.2 |  | 7280 | Wines | 7.20 | 4.46 |
|  | 5600 | Mountain | 7.57 | 5.19 |  | 7350 | Pizza | 7.10 | 4.97 |
|  | 5760 | Outdoors | 8.05 | 3.22 |  | 7460 | French fries | 6.81 | 5.12 |
|  | 7260 | Cake | 7.21 | 5.11 |  | 8120 | Tennis | 7.09 | 4.85 |
|  | 7270 | Ice Cream | 7.53 | 5.76 |  | 8180 | Cliff Diving | 7.12 | 6.59 |
|  | 8030 | Ski jump | 7.33 | 7.35 |  | 8186 | Sky surfing | 7.01 | 6.84 |
|  | 8090 | Gymnastics | 7.02 | 5.71 |  | 8210 | Boat | 7.53 | 5.94 |
|  | 8200 | Water ski | 7.54 | 6.35 |  | 8500 | Gold | 6.96 | 5.6 |
|  |  |  |  |  |  |  |  |  |  |
|  |  |  |  |  |  |  |  |  |  |
| RECOGNITION ONLY (DISTRACTORS) | | | | | | | | | |
| **Negative** | 1112 | Snake | 4.71 | 4.6 | **Negative** | 1200 | Spider | 3.95 | 6.03 |
|  | 1113 | Snake | 3.81 | 6.06 |  | 2110 | Angry face | 3.34 | 5.18 |
|  | 1205 | Spider | 3.65 | 5.79 |  | 2276 | Girl | 2.67 | 4.63 |
|  | 1270 | Roach | 3.68 | 4.77 |  | 2810 | Boy | 4.31 | 4.47 |
|  | 2100 | Angry face | 3.85 | 4.53 |  | 3080 | Mutilation | 1.48 | 7.22 |
|  | 3015 | Accident | 1.52 | 5.9 |  | 3101 | Burnt face | 1.91 | 5.6 |
|  | 3064 | Mutilation | 1.45 | 6.41 |  | 3168 | Mutilation | 1.56 | 6 |
|  | 3160 | Eye disease | 2.63 | 5.35 |  | 3181 | Batter | 2.30 | 5.06 |
|  | 3280 | Dental exam | 3.72 | 5.39 |  | 3261 | Tumor | 1.82 | 5.75 |
|  | 3350 | Child | 1.88 | 5.72 |  | 3266 | Injury | 1.56 | 6.79 |
|  | 9040 | Child | 1.67 | 5.82 |  | 3301 | Injured | 1.80 | 5.21 |
|  | 9041 | Scared child | 2.98 | 4.64 |  | 6311 | Distress. fem. | 2.58 | 4.95 |
|  | 9045 | Native female | 3.75 | 3.89 |  | 8010 | Runner | 4.38 | 4.12 |
|  | 9090 | Exhaust | 3.56 | 3.97 |  | 9007 | Needle | 3.10 | 3.67 |
|  | 9101 | Cocaine | 3.62 | 4.02 |  | 9046 | Family | 3.32 | 4.31 |
|  | 9190 | Woman | 3.90 | 3.91 |  | 9220 | Cemetery | 2.06 | 4 |
|  | 9290 | Garbage | 2.88 | 4.4 |  | 9360 | Empty pool | 4.03 | 2.63 |
|  | 9500 | Porpoise | 2.42 | 5.82 |  | 9440 | Skulls | 3.67 | 4.55 |
|  |  |  |  |  |  |  |  |  |  |
|  |  |  |  |  |  |  |  |  |  |
| **Table S1** continued | | | | | | | | | |
|  |  |  |  |  |  |  |  |  |  |
| **Neutral** | 1670 | Cow | 5.82 | 3.33 | **Neutral** | 2840 | Chess | 4.91 | 2.43 |
|  | 2215 | Neut. Man | 4.63 | 3.38 |  | 2880 | Shadow | 5.18 | 2.96 |
|  | 2280 | Boy | 4.22 | 3.77 |  | 5130 | Rocks | 4.45 | 2.51 |
|  | 2320 | Girl | 6.17 | 2.9 |  | 5390 | Boat | 5.59 | 2.88 |
|  | 5520 | Mushroom | 5.33 | 2.95 |  | 5731 | Flowers | 5.39 | 2.74 |
|  | 5530 | Mushroom | 5.38 | 2.87 |  | 7040 | Dust pan | 4.69 | 2.69 |
|  | 6000 | Prison | 4.04 | 4.91 |  | 7170 | Light bulb | 5.14 | 3.21 |
|  | 6150 | Outlet | 5.08 | 3.22 |  | 7179 | Rug | 5.06 | 2.88 |
|  | 7000 | Rolling | 5.00 | 2.42 |  | 7205 | Scarves | 5.56 | 2.93 |
|  | 7010 | Basket | 4.94 | 1.76 |  | 7217 | Clothes | 4.82 | 2.43 |
|  | 7020 | Fan | 4.97 | 2.17 |  | 7224 | File cabinet | 4.45 | 2.81 |
|  | 7030 | Iron | 4.69 | 2.99 |  | 7233 | Plate | 5.09 | 2.77 |
|  | 7080 | Fork | 5.27 | 2.32 |  | 7237 | Abstract | 5.43 | 3.88 |
|  | 7100 | Fire hydrant | 5.24 | 2.89 |  | 7490 | Window | 5.52 | 2.42 |
|  | 7110 | Hammer | 4.55 | 2.27 |  | 7491 | Building | 4.82 | 2.39 |
|  | 7161 | Pole | 4.98 | 2.98 |  | 7500 | Building | 5.33 | 3.26 |
|  | 7187 | Abstract art | 5.07 | 2.3 |  | 7560 | Freeway | 4.47 | 5.24 |
|  | 7235 | Chair | 4.96 | 2.83 |  | 7830 | Agate | 5.26 | 4.08 |
|  |  |  |  |  |  |  |  |  |  |
| **Positive** | 1460 | Kitten | 8.21 | 4.31 | **Positive** | 1590 | Horse | 7.24 | 4.8 |
|  | 1740 | Owl | 6.91 | 4.27 |  | 1620 | Sprgbok | 7.37 | 3.54 |
|  | 2150 | Father | 7.92 | 5 |  | 2071 | Baby | 7.86 | 5 |
|  | 2530 | Elderly | 7.80 | 3.99 |  | 2080 | Baby | 8.09 | 4.7 |
|  | 2540 | Mother | 7.63 | 3.97 |  | 2270 | Child | 6.28 | 3.15 |
|  | 4180 | Female nude | 6.21 | 5.54 |  | 2510 | Old woman | 6.91 | 4 |
|  | 4490 | Erotic male | 6.27 | 6.06 |  | 4220 | Erotic female | 8.02 | 7.17 |
|  | 4603 | Romance | 7.10 | 4.89 |  | 4520 | Erotic male | 6.16 | 4.8 |
|  | 4650 | Couple | 6.96 | 5.67 |  | 4599 | Romance | 7.12 | 5.69 |
|  | 4660 | Erotic couple | 7.40 | 6.58 |  | 4652 | Couple | 6.79 | 6.62 |
|  | 5001 | Sun flower | 7.16 | 3.79 |  | 4800 | Erotic couple | 6.44 | 7.07 |
|  | 5830 | Sunset | 8.00 | 4.92 |  | 7230 | Turkey | 7.38 | 5.52 |
|  | 7200 | Brownie | 7.63 | 4.87 |  | 7325 | Water melon | 7.06 | 3.55 |
|  | 7330 | Ice cream | 7.69 | 5.14 |  | 7470 | Pan cakes | 7.08 | 4.64 |
|  | 7400 | Candy | 7.00 | 5.06 |  | 7481 | Food | 6.53 | 4.92 |
|  | 7580 | Desert | 7.51 | 4.59 |  | 8080 | Sailing | 7.73 | 6.65 |
|  | 8170 | Sail boat | 7.63 | 6.12 |  | 8190 | Skier | 8.10 | 6.28 |
|  | 8185 | Sky dive | 7.57 | 7.27 |  | 8460 | Runner | 6.40 | 4.55 |

***References:*** *Lang, P.J., Bradley, M.M., & Cuthbert, B.N. (2008). International affective picture system (IAPS): Affective ratings of pictures and instruction manual. Technical Report A-8. University of Florida, Gainesville, FL*

**Supplementary Table S2:** Normative values for valence and arousal. Mean values and SDs of International Affective Picture System (IAPS, Lang et al., 2001) pictures used for this study and category specific comparisons between sets.

|  | Valence | | | Arousal | | |
| --- | --- | --- | --- | --- | --- | --- |
| Picture category | Mean (SD) | | Z (p)^a^ | Mean (SD) | | Z (p)^a^ |
|  | Set A | Set B | A > B | Set A | Set B | A > B |
| ***Perception & Recognition*** | | | | | | |
| **Neg**  (L)  (H) | 3.65 (0.52)  2.57 (0.88) | 3.47 (0.74)  1.93 (0.44) | 0.44 (.659)  1.50 (.133) | 3.86 (0.55)  6.28 (0.68) | 3.90 (0.65)  6.22 (0.64) | 0.09 (.930)  0.09 (.930) |
| **Neu**  (L)  (H) | 4.89 (0.16)  5.03 (0.21) | 4.93 (0.21)  5.14 (0.28) | -0.35 (.724)  -0.57 (.566) | 2.53 (0.40)  3.36 (0.37) | 2.52 (0.31)  3.59 (0.35) | 0.75 (.455)  -1.32 (.185) |
| **Pos**  (L)  (H) | 7.46 (0.74)  7.15 (0.60) | 7.30 (0.53)  6.94 (0.37) | 0.71 (.480)  1.72 (.085)^✢^ | 4.29 (0.69)  5.96 (0.80) | 4.42 (0.41)  5.73 (0.66) | 0.01 (.999)  0.66 (.510) |
| ***Recognition only*** | | | | | | |
| **Neg**  (L)  (H) | 3.66 (0.53)  2.53 (0.98) | 3.35 (0.82)  2.19 (0.87) | 0.71 (.480)  0.53 (.596) | 4.30 (0.35)  5.81 (0.32) | 4.15 (0.68)  5.87 (0.74) | 0.18 (.860)  0.26 (.791) |
| **Neu**  (L)  (H) | 5.04 (0.24)  5.00 (0.70) | 4.90 (0.37)  5.22 (0.34) | 1.06 (.289)  -1.02 (.310) | 2.43 (0.38)  3.38 (0.64) | 2.58 (0.17)  3.48 (0.79) | -1.02 (.309)  0.26 (.791) |
| **Pos**  (L)  (H) | 7.55 (0.43)  7.18 (0.62) | 6.95 (0.61)  7.33 (0.64) | **2.08 (.038)^✻^**  -0.62 (.536) | 4.40 (0.44)  5.83 (0.76) | 4.19 (0.64)  6.10 (0.85) | 0.71 (.480)  -0.57 (.566) |

***Abbreviations:*** Neg - negative, Neu - neutral, Pos - positive, L - low arousal, H - high arousal, SD - standard deviation

***Statistics:*** ^a^ Wilcoxon signed rank test; ^✻^ significant at p < .05 uncorrected for multiple tests; ^✢^ statistical trend at p < .1

Table S3 shows the normative valence and arousal of our IAPS sets as provided by the Center for the Study of Emotion and Attention (https://csea.phhp.ufl.edu/Media.html). Except a significant difference in low arousing positive valence pictures used in the recognition phase only (distracter or new pictures), the sets matched perfectly regarding their valence and arousal characteristics. However, also the mentioned difference pass not the significance threshold if results were Bonferroni or even family-wise error (FEW) corrected for the number of conducted comparisons.

**Supplementary Table 3.** Behavior – Descriptive Statistics.

|  | **Pat** | | **HC** | |
| --- | --- | --- | --- | --- |
|  | **Low** | **High** | **Low** | **High** |
| **Valence: mean rating (SD)** | | | | |
| NEG | 3.89 (0.83) | 2.86 (0.59) | 3.53 (0.57) | 2.19 (0.61) |
| NEU | 5.00 (0.50) | 5.07 (0.54) | 4.98 (0.31) | 5.07 (0.44) |
| POS | 6.56 (0.70) | 6.16 (0.57) | 7.12 (0.45) | 6.75 (0.65) |
| **Arousal: mean rating (SD)** | | | | |
| NEG | 2.50 (0.96) | 4.05 (1.29) | 3.43 (1.20) | 5.53 (1.34) |
| NEU | 1.28 (0.36) | 1.34 (0.27) | 1.65 (0.67) | 2.10 (0.79) |
| POS | 2.10 (1.28) | 2.24 (1.05) | 2.90 (1.59) | 3.55 (1.62) |
| **Error rates: mean in percent (SD)**  Known (encoded) pictures | | | | |
| NEG | 2.1 (6.0) | 3.5 (6.7) | 1.4 (3.8 | 2.1 (6.0) |
| NEU | 2.8 (6.4) | 2.1 (6.0) | 4.2 (6.9) | 2.1 (6.0) |
| POS | 2.1 (4.5) | 2.8 (5.0) | 1.4 (5.5) | 1.4 (5.5) |
| Unknown (distractor) pictures | | | | |
| NEG | 3.5 (6.7) | 5.5 (8.1) | 2.1 (4.5) | 3.5 (5.3) |
| NEU | 6.9 (10.6) | 4.2 (8.9) | 10.4 (13.1) | 3.5 (6.7) |
| POS | 0.7 (2.8) | 4.9 (5.7) | 0.0 (0.0) | 5.5 (7.0) |
| **Response times: mean in seconds (SD)**  Familiar (encoded) pictures | | | | |
| NEG | 1.17 (0.20) | 1.26 (0.31) | 1.12 (0.20) | 1.16 (0.24) |
| NEU | 1.12 (0.18) | 1.16 (0.20) | 1.04 (0.17) | 1.12 (0.14) |
| POS | 1.30 (0.37) | 1.23 (0.28) | 1.09 (0.19) | 1.15 (0.25) |
| Unfamiliar (distractor) pictures | | | | |
| NEG | 1.41 (0.41) | 1.45 (0.46) | 1.33 (0.28) | 1.40 (0.30) |
| NEU | 1.29 (0.41) | 1.27 (0.24) | 1.24 (0.27) | 1.18 (0.24) |
| POS | 1.20 (0.24) | 1.46 (0.38) | 1.23 (0.26) | 1.39 (0.22) |

***Abbreviations:*** *NEG - negative, NEU - neutral, POS - positive, L - low arousal, H - high arousal, SD - standard deviation*
